# An Efficient Chemoselective Reduction of Furan Series Unsaturated Dinitriles

**DOI:** 10.3390/molecules18022212

**Published:** 2013-02-11

**Authors:** Pavel Bobal, Janette Bobalova

**Affiliations:** 1 Department of Chemical Drugs, Faculty of Pharmacy, University of Veterinary and Pharmaceutical Sciences Brno, Palackeho 1/3, 612 42 Brno, Czech Republic; 2 Institute of Analytical Chemistry of the Academy of Sciences of the Czech Republic, v. v. i., Veveří 97, 602 00 Brno, Czech Republic

**Keywords:** chemoselective reduction, regioselective reduction, conjugated olefins, organic hydride compounds, furan ethylenes, unsaturated dinitriles, 2-phenylbenzimidazoline

## Abstract

An efficient reduction of double bonds conjugated with nitrile groups and acid or base sensitive furan rings with 2-phenylbenzimidazoline generated *in situ* has been successfully accomplished with high yields and excellent selectivity. The employed reducing agent was prepared in one step from ordinary chemicals. The other advantages of the presented method include mild and convenient reaction conditions, a benign and cost effective reagent, simple work-up and separation of the products. As this process does neither affect cyano and nitro groups nor furan rings, it is a valuable alternative when metal-catalyzed hydrogenations or borohydride reductions have failed.

## 1. Introduction

Substituted furans are important and ubiquitous structural units present in large number of natural compounds [1], pharmaceuticals [1,2] and have been widely used as synthetic building blocks for the synthesis of many organic materials [3,4]. Their synthesis, however, still remains a challenge for synthetic chemists. The chemoselective reduction of conjugated double bonds with a furan nucleus constitutes a rather troublesome case, as a consequence of the ease of saturation of the heterocyclic moiety [5,6], and the well-known sensitivity of furans to either acids or bases [7,8].

Further, the reduction of substituted propenenitriles to saturated propanenitriles often proceeds with concomitant reduction of the nitrile moiety, polymerization and other side reactions [9]. At present, numerous methods for the reduction of unsaturated nitriles have been developed, including catalytic hydrogenation [10], reductions with organostannic hydrides [11,12], formic acid-triethylamine azeotrope [13], magnesium in methanol [9], sodium borohydride [14,15], sodium cyanoborohydride [16], and many others. Despite the fact that a large group of reducing agents are used for reduction of conjugated unsaturated nitriles, only a limited number might be considered as really selective and accepted as being synthetically useful. To this group belong the so called organic hydride compounds. They can transfer hydrogen to the substrates in the form of hydride anions [17]. These hydride donors are well known in different forms in nature, such as NADH, FADH_2_ and others. In recent years, many biomimetic organic hydride compounds were synthesized mainly containing six-membered heterocyclic systems like Hantzsch esters possessing 1,4-dihydropyridine as a redox active centre [18,19,20,21]. The other group of heterocyclic compounds having hydrogen-donating ability is a group of five-membered heterocycles, such as dihydrobenzazoles and their usefulness as selective reducing agents has been shown [22,23,24,25]. Finally *N,N,N',N',N'',N''*-hexaethylphosphorous triamide was also successfully used for the reduction of 3-(furan-2-yl)-2-cyanopropenenitrile and its thiophene analog [26].

## 2. Results and Discussion

### 2.1. Chemistry

The selective reduction of conjugated C=C bonds with strong electron-withdrawing groups like cyano, nitro or carbonyl groups has been one of the most desirable transformations in organic chemistry. Chemoselective reductions of unsaturated nitriles have attracted much attention for several decades because the reduction products were very important intermediates in organic synthesis. 

The goal of the study was to find a suitable method for the chemoselective reduction of 3-phenyl, 3-(thiophen-2-yl) and 3-(5-substituted furan-2-yl)-2-cyanopropenenitriles **1a**–**g**. The products of the reaction *e.g*. aryl and heteroarylmethylpropanedinitriles, are important intermediates for the synthesis of many biologically active heterocyclic compounds [27].

**Scheme 1:**
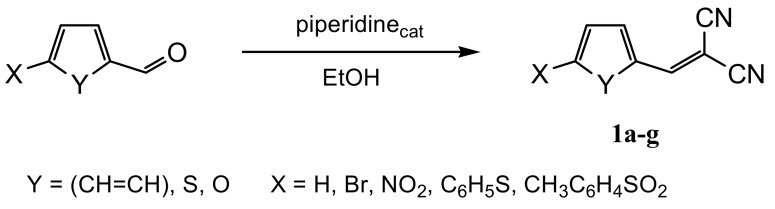
Standard synthesis of 3-phenyl-2-cyanopropenenitrile, 3-(thiophen-2-yl)-2-cyanopropenenitrile and 3-(furan-2-yl)-2-cyanopropenenitriles **1a**–**g**.

The substrates for the reduction reaction, 3-phenyl, 3-(thiophen-2-yl) and 3-(5-substituted furan-2-yl)-2-cyanopropenenitriles **1a**–**g**, were easily synthesized from the corresponding aldehydes and propanedinitrile, via Knoevenagel condensation catalyzed with piperidine in ethanol [28,29] according to Scheme 1. The 5-substituted furan-2-carbaldehydes starting materials were prepared according to procedures described earlier [30,31,32].

The reduction of 3-aryl-2-cyanopropenenitriles is well established [9,10,11,12,13,14,15,16,18,19,20,21,22,23,24,25,33,34,35,36]. A large numbers of processes have been developed for this purpose, among which the most important are catalytic hydrogenation [33,34] and borohydride reduction [35,36]. We have initially started with an investigation of these methods and their application to substituted furan derivatives. In our hands, reduction of 3-phenyl-2-cyanopropenenitrile (**1a**) with sodium borohydride in methanol worked well and we isolated 2-benzylpropanedinitrile (**3a**) [37] in 85% yield. When applied to 3-(thiophen-2-yl)-2-cyanopropenenitrile (**1b**), the result was rather similar. Borohydride reduction of 3-(furan-2-yl)-2-cyanopropenenitrile (**1c**) also afforded product **3c** [38] in a satisfactory 61% yield. The problem arose when this method of reduction was employed for 5-substituted furan olefins **1d**–**g**. In case of 5-bromoderivative **1d** the formation of many side products was observed. Utilization of this process for 5-nitro, 5-phenylsulfanyl and 5-tosylderivatives **1e**–**g** only led to decomposition within a short time and there was no sign of formation of the desired products **3e**–**g**. Fast decomposition was already observed after addition of the first portion of borohydride. The results are summarized in Table 1. All attempts to suppress decomposition by variations of reaction conditions (temperature, ratio of reagents, solvent, reaction time, *etc.*) failed. To overcome this problem catalytic hydrogenation on palladium at atmospheric pressure was tested. The reaction time at atmospheric pressure was quite long (10–48 h) and satisfactory results were achieved in case of phenyl and unsubstituted furyl derivatives. The results showed that the reaction of substituted furan-containing olefins did not proceed cleanly, and the formation of side products, impurities and decomposition was noticed. In the case of 5-nitrofuran derivative **1e** only decomposition was observed, probably caused by instability of the partially reduced nitrofuran function. Sulphur containing compounds **3f**,**3g** were not tested due to anticipated catalyst poisoning. 

**Scheme 2:**
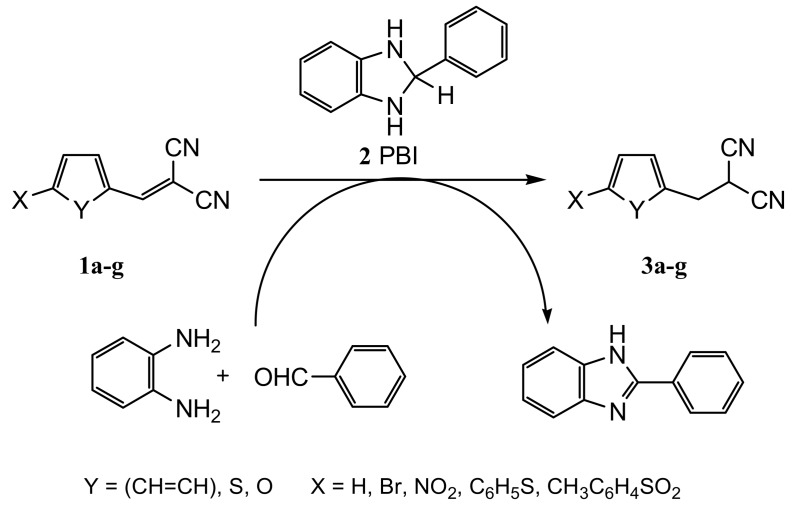
Chemoselective reduction of conjugated double bonds with nitrile groups and furan rings accomplished by using 2-phenylbenzimidazoline.

From these tests it was evident that we must look for milder and more selective reducing agent. Herein, we decided to explore 2-phenylbenzimidazoline (**2** PBI) as an organic hydride compound to test its abilities in reducing electron-deficient olefins with electron-withdrawing substituents (CN groups) of the furan series. 2-Phenylbenzimidazoline (**2** PBI) is unstable in the air and easily oxidized to aromatic 2-phenylbenzimidazole [24]. Therefore, 2-phenylbenzimidazoline is formed *in situ* from the ordinary chemicals (*i.e.*, 1,2-diaminobenzene and benzaldehyde) and is expected to be useful as a convenient, mild and selective agent for the reduction of carbon-carbon double bonds in α,β-unsaturated dinitriles **1a**–**g**. After the reaction of an equimolar amount of 1,2-diaminobenzene and benzaldehyde in ethanol at room temperature in the presence of the same amount of 3-(5-nitrofuran-2-yl)-2-cyanopropenenitrile (**1e**), 2-[(5-nitrofuran-2-yl)methyl]propanedinitrile (**3e**) was obtained in 62% yield. Although a nitro group was inert towards 2-phenylbenzimidazoline, olefinic double bonds conjugated with nitrile groups were reduced efficiently. For optimizing the conditions, several tests with various solvents (MeOH, THF, CH_3_CN, DMF) were performed. When methanol or tetrahydrofuran were used as a solvent the reaction proceeded smoothly, but the reaction time was longer, probably due to the lower solubility of the starting material 3-(5-nitrofuran-2-yl)-2-cyano-propenenitrile (**1e**). Acetonitrile and even more dimethylformamide as reaction solvent catalyzed slow formation of side products. Consequently, ethanol remains as an optimal solvent. When attempts were made to improve the yields and remove the traces of starting α,β-unsaturated dinitrile from the reaction mixtures by increasing the amount of the reagent to 1.2 equivalents, the results showed a disappearance of starting material but the product was a complex mixture. The residues of reducing agent remain in the product. Therefore, all of the experiments were performed with equimolar amounts. In all cases, carbon-carbon double bonds of the 3-(5-substituted furan-2-yl)-2-cyanopropenenitriles were selectively reduced in good yields to give the corresponding 2-[(5-substituted furan-2-yl)methyl]propanedinitriles, while conjugated cyano groups were unaffected, according to Scheme 2.

2-Benzylpropanedinitrile (**3a**), 2-[(thiophen-2-yl)methyl]propanedinitrile (**3b**) and 2-[(furan-2-yl)methyl]propanedinitrile (**3c**) were also synthesized by reduction with 2-phenylbenzimidazoline (**2** PBI) formed *in situ* as well as by NaBH_4_ reduction. All of the results are summarized in Table 1. In this reaction system, the isolation of 2-benzimidazoline (**2** PBI) cannot be achieved, but it is smoothly converted under the reaction conditions to 2-benzimidazole in a practically quantitative yield. After the reaction when ethanol is evaporated, the 2-benzimidazole formed is removed from the reaction mixture by simple filtration of the methylene chloride solution, where this compound is not soluble. The remaining filtrate contains products **3a**–**g** in sufficient (>95%) purity according to HPLC analysis. For analytical purposes the final products were purified by column chromatography and/or recrystallization. 

## 3. Experimental 

### 3.1. General

All chemicals were reagent grade and were purchased from Sigma-Aldrich (St. Louis, MO, USA) and Acros Organics (Geel, Belgium). TLC analysis was performed on precoated 60 F_254_ plates (Merck, Darmstadt, Germany). Compounds were visualized by UV light (254 nm) and evaluated in iodine vapour. HPLC monitoring analyses were performed on an Agilent 1200 series HPLC system equipped with a diode array detection (DAD) system, a quarternary model pump and an automatic injector (Agilent Technologies, Santa Clara, CA, USA). ChemStation Chromatography Software was used for data acquisition. Acetonitrile (HPLC grade, Sigma-Aldrich, 50.0%) and H_2_O (HPLC grade, Sigma-Aldrich, 50.0%) were used as the mobile phase. A Gemini-NX 100 (Phenomenex, Torrance, CA, USA), C18 3 μm, 2.0 × 100 mm chromatographic column was used with a total flow of 0.2 mL/min, an injection volume of 5 μL and a column temperature of 35 °C. A detection wavelength of 210 nm was chosen. The melting points were determined on a Boetius PHMK 05 (VEB Kombinat Nagema, Radebeul, Germany) and are uncorrected. All ^1^H and ^13^C-NMR spectra were recorded in DMSO-*d_6_* solutions at ambient temperature on a Bruker Avance III 400 MHz spectrometer (Karlsruhe, Bruker, Germany, 400 MHz for ^1^H, 100 MHz for ^13^C). Chemical shifts are reported in ppm (δ). Proton chemical shifts in DMSO-*d*_6_ are related to the middle of the solvent multiplet (δ = 2.50). ^13^C-NMR spectra were measured using APT pulse sequences. Carbon chemical shifts are referenced to the middle of the solvent multiplet (δ = 39.5 in DMSO-*d*_6_). Mass spectra were measured using a LTQ Orbitrap Hybrid Mass Spectrometer (Thermo Electron Corporation, Waltham, MA, USA) with direct injection into an APCI source (400 °C) in the positive mode.

### 3.2. Synthesis

#### 3.2.1. Procedure for the Synthesis of 3-(Phenyl)-2-cyanopropenenitrile (**1a**) [29]

To the stirred solution of propanedinitrile (0.66 g, 10 mmol) in absolute ethanol (20 mL) under argon was added benzaldehyde (1.06 g, 10 mmol) followed by 2–3 drops of piperidine. The reaction was refluxed for 2–3 h. The precipitate was formed on cooling the reaction mixture to room temperature. The crude product was filtered and recrystallized from ethanol to yield 3-phenyl-2-cyanopropenenitrile (**1a**) in 75% yield.

#### 3.2.2. General Procedure for the Synthesis of 3-(Thiophen-2-yl) and 3-(5-Substituted Furan-2-yl)-2-cyanopropenenitriles **1b**–**g** [28,29]

To the stirred solution of propanedinitrile (0.66 g, 10 mmol) in absolute ethanol (20 mL) under argon was added 2-thenaldehyde or 5-substituted furan-2-carbaldehyde (10 mmol) followed by 2–3 drops of piperidine. The reaction was stirred for 2–3 h at room temperature. The precipitate was formed during the reaction. The crude product was filtered, washed with cold ethanol and recrystallized from ethanol to yield cyanopropenenitriles **1b**–**g** in 56%–87% yields. 

#### 3.2.3. Procedure for the Synthesis of 2-Benzylpropanedinitrile with NaBH_4_

3-Phenyl-2-cyanopropenenitrile (**1a**, 1.54 g, 10 mmol) was dissolved in methanol (50.0 mL) at room temperature. Then 757 mg (20 mmol) of NaBH_4_ were added in portions. The reaction mixture was stirred at the same temperature for 2 hours, then cooled in ice-bath and 100 ml of water were added. Precipitated crude product was filtrated and recrystallized from ethanol to yield 2-benzylpropanedinitrile in 85% yield. 

#### 3.2.4. General Procedure for the Synthesis of Phenyl-, Thiofen-2-yl and 5-Substituted Furan-2-ylmethylpropanedinitriles **3a**–**g** with 2-Phenylbenzimidazoline (**2** PBI)

Aryl or heteroaryl 2-cyanopropenenitrile (**1a**–**g**, 10 mmol) was dissolved in 96% ethanol (100 mL) under argon. To a stirred mixture was added freshly distilled benzaldehyde (1.06 g, 10 mmol) at room temperature, followed by 1,2-diaminobenzene (1.08 g, 10 mmol). Reaction mixture was stirred at the same temperature and monitored by TLC. After completion of the reaction, ethanol was removed under reduced pressure and the residue was triturated with methylene chloride (30 mL). Precipitated 2-phenylbenzimidazole was filtrated off and washed with methylene chloride (2 × 10 mL). Methylene chloride was removed from combined filtrates under reduced pressure to yield arylmethyl or heteroarylmethylpropanedinitriles **3a**–**g**. The crude products were sufficiently pure for further synthesis. For analytical purposes the products were purified by column chromatography and/or recrystallization (Table 1).

*2-Benzylpropanedinitrile* (**3a**). Yield 83%; M.p. 90–91 °C (M.p. 88–91 °C [37]); ^1^H-NMR (DMSO-*d*_6_), δ: 7.35–7.48 (m, 5H), 4.35 (t, *J* = 7.0 Hz, 1H), 3.37(d, *J* = 7.0 Hz, 2H); ^13^C-NMR (DMSO-*d*_6_), δ: 135.35, 129.94, 129.32, 128.35, 114.57 (2C), 35.01, 24.92.

*2-[(Thiophen-2-yl)methyl]propanedinitrile* (**3b**). Yield 78%; Colourless oil (M.p. 46.5–47 °C [36]); ^1^H-NMR (DMSO-*d*_6_), δ: 7.48 (d, *J* = 5.1 Hz, 1H), 7.23 (d, *J* = 3.2 Hz, 1H), 7.01 (dd, *J* = 5.1, 3.2 Hz, 1H), 4.25 (t, *J* = 6.6 Hz, 1H), 3.56 (d, *J* = 6.6 Hz, 2H); ^13^C-NMR (DMSO-*d*_6_), δ: 134.55, 129.82, 128.15, 127.16, 111.99 (2C), 31.53, 25.95.

*2-[(Furan-2-yl)methyl]propanedinitrile* (**3c**) [38]. Yield 72%; Yellowish oil; ^1^H-NMR (DMSO-*d*_6_), δ: 7.56 (dd, *J* = 1.8, 0.8 Hz, 1H), 6.40–6,35 (m, 2H), 4.64 (t, *J* = 7.0 Hz, 1H), 3.42 (d, *J* = 7.0 Hz, 2H); ^13^C-NMR (DMSO-*d*_6_), δ: 147.02, 143.67, 112.89 (2C), 110.12, 109.21, 29.80, 23.01. 

*2-[(5-Bromofuran-2-yl)methyl]propanedinitrile* (**3d**) [39]. Yield 80%; Colourless oil; ^1^H-NMR (DMSO-*d*_6_), δ: 6.58 (d, *J* = 3.3 Hz, 1H), 6.48 (dt, *J* = 3.3, 0.73 Hz, 1H), 5.15 (t, *J* = 6.2 Hz, 2 H), 3.48 (d, *J* = 6.2 Hz, 1 H); ^13^C-NMR (DMSO-*d*_6_), δ: 150.93, 121.27, 113.57 (2C), 112.78, 112.23, 27.92, 22.42; HR-MS: for C_8_H_6_BrN_2_O [M+H]^+^ calculated 224,9664 *m/z*, found 224,9683 *m/z*.

*2-[(5-Nitrofuran-2-yl)methyl]propanedinitrile* (**3e**). Yield 62%; Brownish oil; ^1^H-NMR (DMSO-*d*_6_), δ: 7.41 (d, *J* = 3.3 Hz, 1H), 6.52 (d, *J* = 3.3 Hz, 1H), 4.92 (t, *J* = 6.5 Hz, 2H), 3.55 (d, *J* = 6.5 Hz, 1H); ^13^C-NMR (DMSO-*d*_6_), δ: 158.62, 150.74, 114.25, 112.87 (2C), 109.39, 27.85, 22.68,; HR-MS: for C_8_H_6_N_3_O_3_ [M+H]^+^ calculated 192.0404 *m/z*, found 192.0438 *m/z*.

*2-{[(5-(Phenylsulfanyl)furan-2-yl]methyl}propanedinitrile* (**3f**). Yield 79%; Yellowish oil; ^1^H-NMR (DMSO-*d*_6_), δ: 7.58–7.39 (m, 5H), 6.51 (d, *J* = 3.3 Hz, 1H), 6.44 (d, *J* = 3.3 Hz, 1H), 5.01 (t, *J* = 6.5 Hz, 2H), 3.52 (d, *J* = 6.5 Hz, 1H);^13^C-NMR (DMSO-*d*_6_), δ: 154.27, 142.15, 135.61, 131.16, 129.56, 125.58, 113.86 (2C), 112.91, 112.35, 27.21, 21.91; HR-MS: for C_14_H_11_N_2_OS [M+H]^+^ calculated 255.0587 *m/z*, found 255.0559 *m/z*. 

*2-({5-[(4-Methylphenyl)sulfonyl]furan-2-yl}methyl)propanedinitrile* (**3g**). Yield 85%; M.p. 65–65 °C; ^1^H-NMR (DMSO-*d*_6_), δ: 7.74 (d, *J* = 8.0 Hz, 2H), 7.37 (d, *J* = 8.0 Hz, 2H), 6.56 (d, *J* = 3.4 Hz, 1H), 6.12 (d, *J* = 3.4 Hz, 1H), 4.89 (t, *J* = 6.6 Hz, 2H), 3.63 (d, *J* = 6.6 Hz, 1H), 2.32 (s, 3H); ^13^C-NMR (DMSO-*d*_6_), δ: 155.21, 148.38, 145.95, 138.22, 130.14, 129.21, 112.33 (2C), 110.82, 107.19, 27.43, 22.19, 21.30; HR-MS: for C_15_H_13_N_2_O_3_S [M+H]^+^ calculated 301.0641 *m/z*, found 301.0657 *m/z*.

## 4. Conclusions

In summary, we have demonstrated that the reductions of α,β-unsaturated dinitriles possessing substituted furan rings could be achieved in high yields and chemoselectivity with 2-phenyl-benzimidazoline (**2**, PBI) formed *in situ*. The easy work-up and separation of products make this reinvented reagent a promising alternative reducing agent in organic synthesis. The reduction of furan compounds **1a**–**g** to **3a**–**g** using PBI in ethanol was compatible with various substitution patterns and, in the limited cases examined, with other functional groups (Table 1). A particular advantage of this method over borohydride reduction and catalytic hydrogenation was the regioselective reduction of a conjugated double bond in the presence of groups sensitive to reduction conditions or deactivating the mostly used transition metals like palladium. The principal side reactions were not observed. 

## Figures and Tables

**Table 1:** Reaction of 3-phenyl, 3-(thiophen-2-yl) and 3-(5-substituted furan-2-yl)-2-cyanopropenenitriles **1a**–**g** with 2-phenylbenzimidazoline (**2**, PBI) generated *in situ* and with NaBH_4_.

Entry	Substrate	Product	Yield *^a^* (%)	Time (h)
**1a**	Y = (CH=CH), X = H	**3a**	83 *^b^* (85) *^c^*	12 *^b^* (2) *^c^*
**1b**	Y = S, X = H	**3b**	78 *^b^* (69) *^c^*	8 *^b^* (2) *^c^*
**1c**	Y = O, X = H	**3c**	72 *^b^* (61) *^c^*	8 *^b^* (2) *^c^*
**1d**	Y = O, X = Br	**3d**	80 *^b^* (-) *^c,d^*	8 *^b^* (1) *^c^*
**1e**	Y = O, X = NO_2_	**3e**	62 *^b^* (-) *^c,e^*	6 *^b^* (1) *^c^*
**1f**	Y = O, X = C_6_H_5_S	**3f**	79 *^b^* (-) *^c,e^*	12 *^b^* (1) *^c^*
**1g**	Y = O, X = CH_3_C_6_H_4_SO_2_	**3g**	85 *^b^* (-) *^a,e^*	8 *^b^* (1) *^c^*

*^a^* isolated yield; *^b^* results obtained with PBI in ethanol; *^c^* results obtained with NaBH_4_ in methanol; *^d^* partial decomposition; *^e^* decomposition.
